# Impact of dental school critical thinking demonstrations carryover to practice: Survey of 5‐year graduates

**DOI:** 10.1002/jdd.13695

**Published:** 2024-08-23

**Authors:** David C. Johnsen, Leonardo Marchini, John Syrbu, Brian J. Howe, Jennifer E. Hartshorn, Jhanvi Desai, Azeez Butali, Wei Shi

**Affiliations:** ^1^ Pediatric Dentistry, Dental Science Building University of Iowa Iowa City Iowa USA; ^2^ Preventive and Community Dentistry, College of Dentistry Iowa City USA; ^3^ Family Dentistry, College of Dentistry Iowa City Iowa USA; ^4^ Iowa Oral Health Research Institute, College of Dentistry Iowa City Iowa USA

**Keywords:** critical thinking, emulation learning model, learning outcomes, practice application

## Abstract

**Introduction:**

Little literature exists on graduates’ application to practice for explicit critical thinking skills learned in dental school.

**Purposes:**

Discern the (1) degree to which graduates apply explicit critical thinking skillsets in practice; (2) degree of adaptation of critical thinking skillsets to practice; (3) frequency of use for critical thinking skillsets in practice; and (4) perceptions to improve critical thinking learning guidance in dental school.

**Methods:**

Five critical thinking exercises/skillsets were selected that had been in place over 5 years with at least one paper: geriatrics, treatment planning, technology decision making, ethics, evidence‐based dentistry; each followed concepts from an emulation model in critical thinking. Electronic survey administered in 2023/2024 to alumni graduated in the last 5 years.

**Results:**

Of 98 (from 320 distributed) returned, 56 completed the entire survey. Dental school experiences positively influenced use of critical thinking skills in practice. On a five‐point scale, mostly 4s and 5s were reported for “…benefit your thinking.” Fifty‐three percent reported “using ideas from the exercise and developed my own thought processes,” 35% reported “using the thought process largely as offered in the college” and 5% reported “do not use the exercise.” Sixty percent reported using the skillsets hourly or daily. With minor variations all skillsets were reported positively for use in practice.

**Conclusions:**

A positive influence of critical thinking skills was gained from the college experience with explicit positive impact for each of the five critical thinking experiences. The questions may be a model for future follow‐up studies of explicit dental school critical thinking exercises.

## 1 INTRODUCTION

Critical thinking is an essential skill^1^ and an essential component for the successful practice of dentistry.^2^ There is no national consensus on defining the learning outcome, guiding learning, and assessing performance for critical thinking skills.^3^ The literature is sparse on learning outcomes for critical thinking skills, sparser on outcomes‐based assessment of critical thinking, and essentially nonexistent on graduates’ application of explicit critical thinking skills to practice resulting from experiences in dental school—a reason for this project. The general purpose of the project is to gain a window into the carryover of critical thinking skillsets from dental school into practice. As a baseline, the theoretical basis for critical thinking exercises (skillsets) is offered with over 10 peer reviewed examples for a learning model based on emulation of the intended activity. We are not aware of another reported learning model for critical thinking with explicit peer reviewed examples. The question is how and how many graduates carry explicit dental school experiences for critical thinking into practice. A learning outcome for critical thinking skills can be the thought process of the expert.^2^, ^4^, ^5^

The following paragraphs offer in sequence a learning model developed from ideas in the education literature with application to a series of critical thinking skillsets; next is use of the umbrella term “patient‐based, student‐led demonstrations of thinking and judgment”; next is the importance of following experiences from school into practice; next is the selection of five critical thinking skillsets to survey graduates on use in practice; and finally is a hypothesis with purposes of the project.

A learning model for critical thinking was developed from ideas in the education literature. With little literature on learning outcomes for critical thinking and no explicit models for critical thinking guidance and assessment in 2010, ideas on emulation from the education literature were synthesized and first published in the dental education literature in 2012.^4^, ^6^ The concepts on emulation were derived from ideas in the education literature on “complex thought processes”—interpreted to reflect critical thinking.^5^ A synthesis of ideas into a prototype or explicit learning guide for critical thinking was initially absent at that time. A basic premise is that a designated thought process is the learning outcome, the learning guide, and the assessment instrument. The goal is to guide what the student is to do in their thought processes—specifically to demonstrate the designated thought process with their patient. The designated thought process is derived from master clinicians and master teachers succinctly enough for the novice to apply to the next patient. With no reference point for a “perfect” thought process, the designated thought process is inherently imperfect initially with continual adjustments.

**FIGURE 1:**
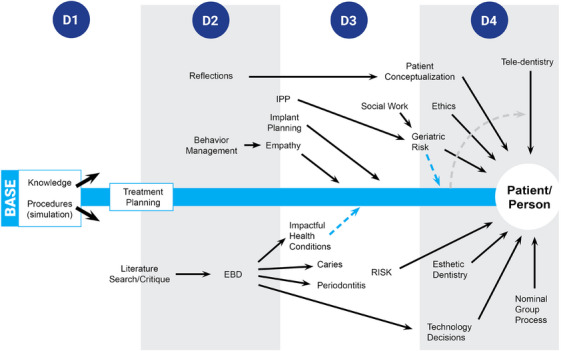
Schematic for patient‐based, student‐led demonstrations of thinking and judgment across 4 years of dental school. While individual exercises were developed separately, a network is formed with individual learning outcomes reinforcing the general culture of critical thinking. All share the concept of emulating the intended thought process with the thought process becoming the learning outcome, the learning guide, and assessment instrument. EBD, evidence‐based dentistry; IPP, interprofessional practice.

Concepts on emulation have been used to develop and describe critical thinking exercises (Figure 1) in treatment planning,^7^ literature search and critique,^8^ caries risk assessment,^9^ ethics,^10^ geriatric dentistry,^11^ interprofessional practice,^12^ social work,^10^ empathy projection,^13^ conceptualizing the next patient interaction,^14^ evidence‐based dentistry,^15^ technology decision making,^16^, ^17^ and teledentistry.^18^ In year 1 of dental school, students begin learning the thought processes for skillsets in critical thinking. While students have limited experience, beginning students are guided and judged based on their thought processes (e.g., treatment planning) more than on the treatment alternatives they develop. We are not aware of another model to develop explicit critical thinking skill sets that has been peer reviewed. With no place to start, the emulation model described below is considered a starting point for active solicitation for other critical thinking learning models. We hope this paper serves as an active invitation to develop addition models for critical thinking in the patient setting as there is now largely a void.^2^

The umbrella term “patient‐based, student‐led demonstrations of thinking and judgment” was selected to try to encompass the theme of the collection of exercises. Each of the exercises is patient or case based; “student led” is key in critical thinking demonstration with the student applying the designated thought process to the patient or case; in each of the exercises, the student applies a designated thought process to a patient/case; in each case, structured thinking and judgment are called for as there are no preconceived “right answers.”

An emulation model for critical thinking was derived from ideas on “complex thought processes” in the education literature.^4^, ^5^ The basic concepts are (1) Engage the student in multiple situations calling for critical thinking; the more such situations, the easier the student will adapt to the next one.^19^, ^20^ Figure 1 shows the context for the critical thinking skillsets selected; while five individual critical thinking skillsets were sampled with graduates, they did not happen in isolation and are synergistic. For example, skillsets for social work and interprofessional practice were each developed separately; seeing an opportunity to consolidate three skillets, social work, and interprofessional practice skillsets were combined into geriatrics (Supporting Information). Questions from social work and interprofessional practice have also been incorporated into other skillsets involving risk assessment. (2) Emulate the intended activity, with the more direct the emulation the greater the validity.^5^, ^21^, ^22^, ^23^, ^24^, ^25^, ^26^, ^27^ The concept of novice to expert comes into play as the expert has highly structured but intuitive thought processes and the novice needs explicit structure.^4^, ^16^, ^28^ (3) Gain agreement of faculty on the content, application, and assessment for reliability/reproducibility.^24^, ^26^, ^29^ (4) Use the same instrument to guide leaning and assess performance.^25^, ^27^, ^30^ Thus, the thought process becomes the learning outcome, the learning guide and the assessment instrument as described in the examples above.^4^, ^6^

Assessment is based on mastery of the thought process as applied to the patient or case. One definition of critical thinking is the art of analyzing one's thought process with the intent of improving it.^31^ Since an “art” has limitations for in quantification, assessments in the above exercises involve an objective component—did the student systematically apply each step in the thought process/learning guide?—and a subjective component—did the student grasp the concept for each step? The objective and subjective components are described in referenced examples.^7^, ^8^, ^9^, ^10^, ^11^, ^12^, ^13^, ^14^, ^15^, ^16^, ^17^, ^18^

Five patient‐based, student‐led demonstrations of thinking and judgment were selected from the 17 critical thinking skillsets in Figure 1. Selection was based on (1) exercises that followed an emulation model for critical thinking just described, (2) had been in operation for at least 5 years, (3) had at least one published paper to describe details for each learning outcome, learning guide, and the logistics of the exercise. The exercises were: geriatrics,^11^ treatment planning,^7^ technology decision making,^17^ ethics,^10^ and evidence‐based dentistry.^15^ Each has a reference with details of the learning outcome and the learning guide. (As an example, for geriatrics the learning guide is shown from a previous papers in Supporting Information.^10^, ^11^, ^12^, ^16^) Other exercises have been in operation less than 5 years or have not yet had a peer reviewed publication. We look forward to sampling other exercises when they meet these criteria. We are not aware of any critical thinking exercises that have been followed into practice. The questions selected could serve as a pilot model to follow critical thinking exercises into practice.

Each of the demonstrations has a different profile. Treatment planning has been in place the longest, and it is used in all clinical departments with variations from a standard learning guide. The current geriatrics format has been in place for about 8 years with a didactic and clinical component going back over 30 years. Evidence‐based dentistry has been in place for over a decade with the demonstration of evidence‐based dentistry concepts included into at least four different department demonstrations. Ethics demonstration has been in place for over two decades. Technology decision making has been in place for about 8 years and is the only demonstration without a specific didactic component. Three of the demonstrations—treatment planning, ethics, and evidence‐based dentistry—are reinforced in the D4 comprehensive clinic using a nominal group process approach.^32^, ^33^ Nominal group process is assignment of a daily performance score from a table of general criteria, briefly described: For each patient visit, the faculty member refers to a table of written general criteria (“criteria for daily feedback for professional development”) and assigns a mark of S (surpassed expectations), M (met expectations), or F (failed to meet expectations) in each of six domains: treatment planning/sequencing/execution; integration of evidence‐based dentistry; technical skills; patient and appointment management; self‐evaluation/independence; professionalism/ethical behavior.

With little literature on learning outcomes for critical thinking and essentially none on carryover to practice for explicit critical thinking exercises in dental schools, a hypothesis is that graduates will take part of what they learned in school and part from practice. The proposition is that determining the degree to which graduates take critical thinking skillsets to practice will allow refinement of learning guidance for critical thinking in school. A plan for refining current critical thinking skillsets will depend on input regarding current carryover to practice. The purposes of the project are to (1) discern the degree to which graduates apply explicit critical thinking skillsets in practice; (2) discern the degree of adaptation of critical thinking skillsets to practice; (3) discern the frequency of use for critical thinking skillsets in practice; and (4) glean perceptions to improve critical thinking learning guidance in dental school.

## 2 METHODS

Approval was gained from the Institutional Review Board (IRB) at the Uinversity of Iowa, #202309644.

This is a cross‐sectional study using a survey. Topics were selected from critical thinking exercises that had been operational for at least 5 years. Topics/categories selected were geriatrics, technology decision making, ethics, treatment planning, and evidence‐based dentistry. Geriatrics was not required for all students for the entire 5‐year period, so a “not applicable” category was included.

Five referees (teaching faculty experienced with surveys and with experience in peer reviewed publication) were asked to rate each item on a four‐point Likert scale, ranging from 1 (not relevant) to 4 (very relevant). A content validity index (CVI) was obtained by dividing the number of referees who choose options 3 and 4 into the total number of referees. The accepted CVI is 0.80.^34^ A low CVI indicates that certain items should be eliminated or revised to establish sufficient content validity.^34^ One question was eliminated. Remaining questions received scores of 0.8 or 1. The CVI was judged adequate to proceed.

For the first purpose of the project, “Discern the degree to which graduates apply explicit critical thinking skillsets in practice.” Question 1 and Question 3 were intended to gain perceptions into that purpose. For Question 1, “On a scale of 1 (not much) to 5 (a great deal), to what extent did your experience in the college of dentistry help you to implement or develop critical thinking skills?” For Question 3 “Were exercises offered at the college a benefit in your thinking for the following areas?” From **1** to **5** with **1** being “little or no influence on my current thinking” and **5** being “strongly influenced my current thinking.” For the second purpose of the project, “Discern the degree of adaptation of critical thinking skillsets to practice.” Question 4 was intended to gain perceptions into that purpose: “Which of the following is the best description of your practice activities?”: 1 being “I do not use the ideas from the exercise in the college”; 2 being “I use ideas from the exercise in the college and developed my own thought process”; and 3 being “I use the thought process largely as offered in the college.” For the third purpose, “Discern the frequency of use for critical thinking skillsets in practice.” Question 5 was intended to gain perceptions into that purpose: “How often do you use your thinking and judgment for each area listed below? Hourly, daily, weekly, monthly, not at all.” Question 2 was developed to see if students applied critical thinking skills in a more structured or more intuitive fashion. The results of this question could give perceptions into use of the skillset in dental school by knowing how graduates would use the skillset in practice.

Once the questions were determined, logistical tasks included: draft a cover letter for potential participants, collaborate with biostatistics colleagues, pilot with seven recent graduates, gain IRB exemption, interact with the College Office of Education, acquire a list of DDS graduates from the last 5 years from the University Alumni Office, convert the survey to Qualtrics, distribute the survey via email, retrieve results, analyze results, develop a report addressing project purposes, share the report with appropriate constituencies. Three separate emailings were conducted, each with a follow‐up for a total of six emailings. A descriptive analysis reporting the frequencies of responses to each survey question was performed. The Fisher's exact test and the chi‐square test were used of data analysis.

## 3 RESULTS

**FIGURE 2:**
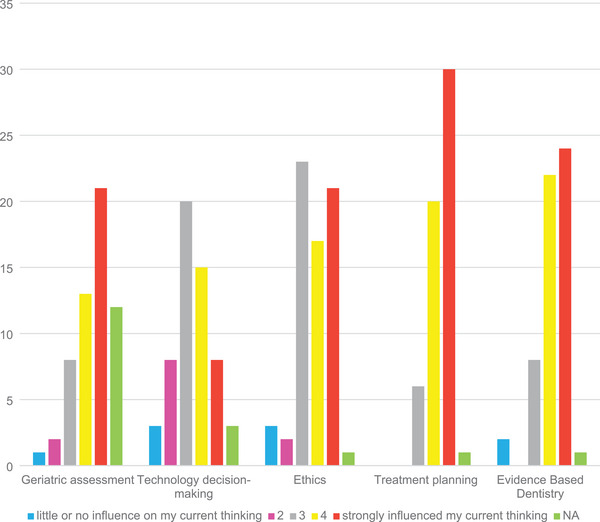
Question 3. Were exercises offered at the college a benefit in your thinking for the following areas?

**FIGURE 3:**
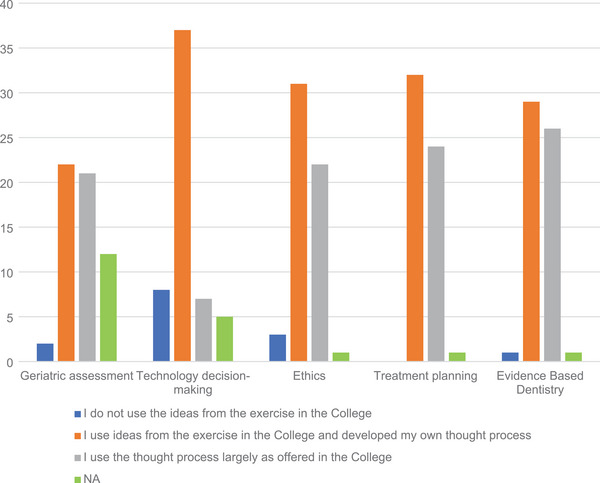
Question 4. Which of the following is the best description of your practice activities?

Ninety‐eight surveys were returned out of 320 for a return rate of 31%. From the 98 returned surveys, 56 completed the entire survey. For the first purpose of the project, “Discern the degree to which graduates apply explicit critical thinking skillsets in practice.” Responses to Question 1 “On a scale of 1 (not much) to 5 (a great deal), to what extent did your experience in the college of dentistry help you to implement or develop critical thinking skills?,” are reported in Table 1 (Question 1). Results were skewed to the positive with two responding with a 1 (“not much”) and 74 (76%) responding a 4 or a 5 (“a great deal”). Responses to Questions 2, 3, 4, and 5 with more specific perspectives on critical thinking were consistent with the general positive responses for Question #1. Continuing with the first purpose of the project, “Discern the degree to which graduates apply explicit critical thinking skillsets in practice.” Responses to Question 3, “Were exercises offered at the college a benefit in your thinking for the following areas? From **1** to **5** with **1** being ‘little or no influence on my current thinking’ and **5** being ‘strongly influenced my current thinking,’” responses are shown in Figure 2. An overall escalating sequence was found from a score of 1 (“little or no influence”) with 3%, 4% with a score of 2 with an increase at the score of 3 with 21% up to the score of 5 (“strongly influenced my current thinking”) with 39%. Combining responses for 4 and 5 versus combined responses for 1 and 2 (3 was omitted), using the Fisher's exact test, the *p* value is <0.001. For the second purpose of the project, “Discern the degree of adaptation of critical thinking skillsets to practice.” Question 4, “Which of the following is the best description of your practice activities?”: 1 being “I do not use the ideas from the exercise in the college.”; 2 being “I use ideas from the exercise in the college and developed my own thought process.”; and 3 being “I use the thought process largely as offered in the college.”, results are shown in Figure 3. The lowest score was for the recording of 1 with 5% to the largest for the recording of 2 with 53%, followed by the recording of 3 with 35%. Comparing responses for “not used” versus the other two responses combined, the Fisher's exact test had a *p* value of 0.0047, *p* value is <0.01 (for response 1 [“not used”] vs. response 2 [“developed own”] plus response 3 [“largely as offered in the college”]).

**TABLE 1:** Question 1. On a scale of 1 (not much) to 5 (a great deal), to what extent did your experience in the college of dentistry help you to implement or develop critical thinking skills?

1	2
2	4
3	18
4	41
5	33
**Total**	**98**

**FIGURE 4:**
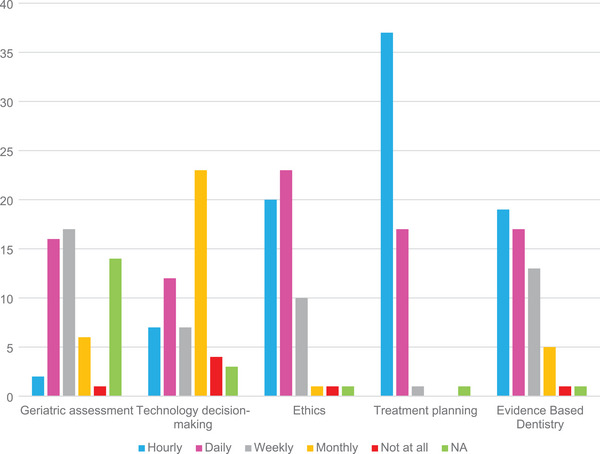
Question 5. How often do you use your thinking and judgment for each area listed below?

**FIGURE 5:**
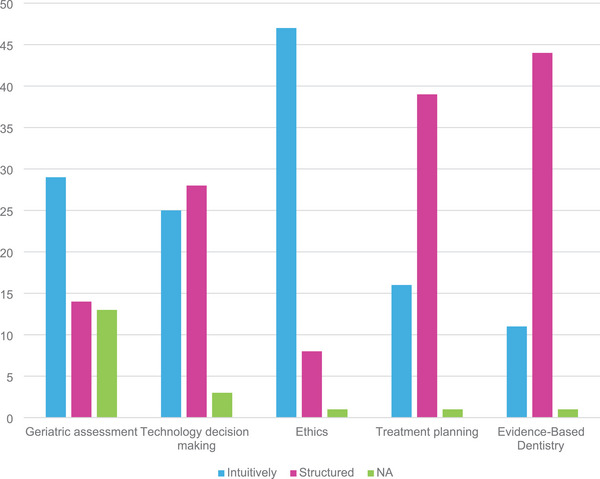
Question 2. Do you make decisions and take actions in the following areas more intuitively or more with a structured guide?

For the third purpose, “Discern the frequency of use for critical thinking skillsets in practice.” Question 5, “How often do you use your thinking and judgment for each area listed below? Hourly, daily, weekly, monthly, not at all,” results are shown in Figure 4. Scores were tilted toward hourly and daily use with treatment planning having the highest percentage of entries for #1 (hourly) followed by evidence‐based dentistry and ethics. Sixty percent recorded hourly or daily use and seven recordings (3%) reported not using a skillset at all. Comparing responses for recordings “hourly” plus “daily” versus “monthly” plus “not at all,” the Fisher's exact test had a *p* value of <0.001. For **Question #2**, “Do you make decisions and take actions in the following areas more intuitively or more with a structured guide?,” results are shown in Figure 5. Geriatric assessment and ethics had the highest percent that chose “intuitive” with 29 and 47, respectively, while treatment planning and evidence‐based dentistry were the opposite with 39 and 44 respectively choosing “More with a structured guide.” Technology decision making was in between. Combining geriatrics and ethics versus treatment planning combined with evidence‐based dentistry, the chi‐square statistic is 58.25; the *p* value is <0.001. The “not applicable” category was included because geriatrics was not required of all students for all the last 5 years. The largest “not applicable” responses were expected for geriatrics with respective questions having 13, 12, 12, and 14 for respective questions. Remaining categories had lower numbers of “not applicable.”

## 4 DISCUSSION

Survey results are interpreted with caution due to the modest response rate. The theoretical learning model based on emulation has held up for over a decade with peer reviewed publications, but without a follow‐up into practice for any critical thinking skillsets until now. Results are interpreted to show a positive overall influence of critical thinking skills gained from the college experience and taken into practice. For Purpose #1, “Discern the degree to which graduates apply explicit critical thinking skillset in practice,” results are interested to show an explicit positive impact of college experiences for each of the five critical thinking experiences (patient‐based, student‐led demonstrations of thinking and judgment); for Purpose #2, “Discern the degree of adaptation of critical thinking skillsets to practice,” results are interpreted to show a trend for each of the experiences to influence the thought processes of the practitioner or to be used largely as offered by the college; for Purpose #3, “Discern the frequency of use for critical thinking skillsets in practice,” results are interpreted to show a trend for hourly or daily use of the skills. In no example did any trend indicate disregard or nonapplication of critical thinking skills offered by the college. While the experiences engaging students in critical thinking are before graduation, the impact is interpreted to go beyond graduation.

Results showing application to practice for each of the five skillsets are interpreted to support the concept that a robust set of critical thinking experiences is more than linear in the effect of students applying critical thinking in practice. Treatment planning has the most widespread application across all 4 years and across departments; technology decision making has the least with a single exercise as part of a practice management course. Despite the wide array of exercise designs beyond a format following a basic model emulating the intended activity, each experience is interpreted to have an impact beyond graduation.

From a methodology standpoint, the basic emulation model is reinforced: offer multiple experiences reflecting critical thinking^4^, ^5^, ^6^; emulate the intended activity (succinctly derive the thought process of the master clinician to serve as the learning outcome, the learning guide, and the assessment instrument), with the more direct the emulation the greater the validity; gain agreement of faculty on content, application, and assessment for reliability; use the same instrument to guide learning and assess performance.^4^, ^5^, ^6^ These results are interpreted as reinforcement of the emulation approach for graduates’ use of critical thinking skills gained in dental school continuing after graduation.

A comment on the cultural component of critical thinking seems in order. We submit that a culture of inquiry, rigor, respect, and role modeling will enhance a culture of critical thinking and that a culture of critical thinking will enhance a culture of inquiry, rigor, respect, and role modeling. This parallels the ideas of David Hume who said we get our ethics from our feelings more than our reasoning.^35^, ^36^ Such a connection would be difficult to prove, especially since critical thinking learning outcomes alone have little literature. The significance of the work starts with the value placed on critical thinking and the value placed on the transfer of critical thinking skills to practice. This work shows promise on the transfer to practice of explicit critical thinking skills learned in dental school.

The project has several limitations. The return rate limits the strength of conclusions despite the trends which are statistically significant. Without critical thinking learning models for comparison, the current set of questions is a first attempt to follow‐up with graduates on application of critical thinking skillsets to practice. A qualitative approach, such as focus groups, might be used in the future to gain more perceptions about *how* alumni have applied the critical thinking skillsets learned at school in their practices, and what changes did they make in the skillsets.

## 5 CONCLUSIONS

Critical thinking is essential for the successful practice of dentistry. The theoretical basis with over 10 examples for a learning model is offered based on emulation of the intended activity. We are not aware of another reported learning model for critical thinking with explicit peer reviewed examples. The question is how and how much graduates carry explicit dental school experiences for critical thinking into practice. Selecting five critical thinking experiences from dental school, recent graduates were surveyed on how and how much of five critical thinking experiences were carried into practice. The skillsets were geriatrics, treatment planning, technology decision making, ethics, evidence‐based dentistry. Critical thinking experiences that were selected had been operational for at least 5 years and had at least one paper published describing operational aspects. Results are interpreted to show that students carried each of the five critical thinking skillsets into practice. This survey model may serve for future follow‐up surveys. We are not aware of another follow‐up for explicit critical thinking experiences from dental school into practice. Future work can follow graduates use of additional critical thinking skillsets into practice. With the lack of leaning outcomes and learning models for critical thinking, an agenda item for the dental education community can be the development of such outcomes and models.^2^, ^37^, ^38^

## Supporting information

Supporting Information
