# Supplementary material for: Impact of dental school critical thinking demonstrations carryover to practice: Survey of 5‐year graduates
Source: J Dent Educ. 2024 Aug 23;89(1):107–15. doi: 10.1002/jdd.13695 (PMC11783349; doi:10.1002/jdd.13695)
Supplement: Supplementary file 1 — Supporting Information [file JDD-89-107-s001.docx]

**Supplement 1:**

One sample learning guide is offered from the list of references. The Geriatrics learning guide represents a thought process for a Geriatrics patient leading to alternatives more than “right” answers. The student applies each step of the thought process to their patient. For Geriatrics, the thought processes for social work and Interprofessional are incorporated. References offer background for the exercises and details.^10,11,12,16^

Table: Learning guide for the patient-based, student-led demonstration of thinking and judgment for Geriatrics

Basic data gathering

General health conditions

Social support

Oral conditions

Interprofessional perspectives

Patient preferences?

Prioritize health conditions (What can kill them first?)

Patient problems drug related?

Patient capacity to subscribe to recommended treatment?

Barriers to care?

Transportation Power of attorney

Finances Mobility

Diet controlled/not controlled?

Do we have all the data?

Which data are most important? Why?

What if we do nothing – 6 months? 5 Years?

What is the patient’s risk?

Minimum risk/Minimum disease High risk/progressing disease

High risk/minimum disease Destruction has happened

Treatment alternatives?

Specific treatment with rationale?

Prognosis?

Communication plan?

Self-assess

Disease engagement level: Disease Consequences? Disease? Person?
